# LDL-Induced Impairment of Human Vascular Smooth Muscle Cells Repair Function Is Reversed by HMG-CoA Reductase Inhibition

**DOI:** 10.1371/journal.pone.0038935

**Published:** 2012-06-12

**Authors:** Teresa Padró, Roberta Lugano, Maisa García-Arguinzonis, Lina Badimon

**Affiliations:** 1 Cardiovascular Research Center (CSIC-ICCC), Barcelona, Spain; 2 Biomedical Research Institute Sant-Pau (IIB-Sant Pau), Barcelona, Spain; 3 CiberOBN, Institute Carlos III, Barcelona, Spain; 4 Autonomous University of Barcelona, Barcelona, Spain; Brigham and Women's Hospital, Harvard Medical School, United States of America

## Abstract

Growing human atherosclerotic plaques show a progressive loss of vascular smooth muscle cells (VSMC) becoming soft and vulnerable. Lipid loaded-VSMC show impaired vascular repair function and motility due to changes in cytoskeleton proteins involved in cell-migration. Clinical benefits of statins reducing coronary events have been related to repopulation of vulnerable plaques with VSMC. Here, we investigated whether HMG-CoA reductase inhibition with rosuvastatin can reverse the effects induced by atherogenic concentrations of LDL either in the native (nLDL) form or modified by aggregation (agLDL) on human VSMC motility. Using a model of wound repair, we showed that treatment of human coronary VSMC with rosuvastatin significantly prevented (and reversed) the inhibitory effect of nLDL and agLDL in the repair of the cell depleted areas. In addition, rosuvastatin significantly abolished the agLDL-induced dephosphorylation of myosin regulatory light chain as demonstrated by 2DE-electrophoresis and mass spectrometry. Besides, confocal microscopy showed that rosuvastatin enhances actin-cytoskeleton reorganization during lipid-loaded-VSMC attachment and spreading. The effects of rosuvastatin on actin-cytoskeleton dynamics and cell migration were dependent on ROCK-signalling. Furthermore, rosuvastatin caused a significant increase in RhoA-GTP in the cytosol of VSMC. Taken together, our study demonstrated that inhibition of HMG-CoA reductase restores the migratory capacity and repair function of VSMC that is impaired by native and aggregated LDL. This mechanism may contribute to the stabilization of lipid-rich atherosclerotic plaques afforded by statins.

## Introduction

Atherosclerotic lesions with a large lipid-necrotic core and a thin fibrous cap are the most prone to rupture [1] triggering the acute ischemic syndromes. Compared with intact caps, those ruptured are usually thinner, and contain higher amount of infiltrated lipids and a paucity of smooth muscle cells (VSMC) [2], the only vascular resident cells that synthesize extracellular matrix components required for the tensile strength of the fibrous cap of the plaques. In asymptomatic silent disease, plaques are healed by VSMC that accumulate at the sites of rupture, where they secrete an extracellular matrix rich in glycosaminoglycans and type III collagen [3]–[5]. VSMC number is determined by the net effect of VSMC proliferation, migration, and death or apoptosis. Cell migration is associated with dynamic remodeling of the actin-myosin cytoskeleton [6], which is dependent on the phosphorylation/dephosphorylation balance of the myosin regulatory light chain (MRLC) [7]–[8].

A key pathogenic event in the development of atherosclerosis is the retention of colloidal atherogenic lipoproteins, primarily low density lipoprotein (LDL), in the arterial intima. This retention occurs when LDL bind to the extracellular matrix proteoglycans, as versican [9]–[11] that induces LDL aggregation (agLDL) and leads to dysfunction of the vascular resident cells [12]–[13]. agLDL upregulate the expression of low-density lipoprotein receptor-related protein 1 (LRP1) [14]–[15], which in turn internalizes significant amounts of cholesteryl esters from agLDL contributing to the transformation of VSMC into lipid loaded cells [15]–[17]. In previous studies we have demonstrated that agLDL decrease phosphorylated-MRLC (P-MRLC) levels and impairs migration and wound repair in VSMC [18]–[19]. These effects could contribute to the development of soft-high risk plaques with decreased VSMC accumulation.

Statins, inhibitors of 3-hydroxy-methylglutaryl coenzyme A (HMG-CoA) reductase, are widely used cholesterol-lowering drugs [20]–[21] that significantly reduce the presentation of cardiovascular events in patients either with or without previous coronary heart disease [22]–[26]. Vascular remodelling and stabilization of vulnerable plaques are presumed to be major contributors in these beneficial effects. To this respect, intravascular ultrasound imaging (IVUS)-end point studies with statins have shown that they reduce atherosclerotic plaque burden in treated patients [27] and the analysis by magnetic resonance imaging (MRI) of aortic and carotid artery plaques of patients treated with simvastatin have shown that statins reduced the size of the lesions and the thickness of the arterial wall without changes in the lumen size [28]. Also, recent clinical studies reported changes in carotid artery morphology in terms of increasing echogenicity and fibrous tissue content as an effect of statins [29]–[33].

**Figure 1:**
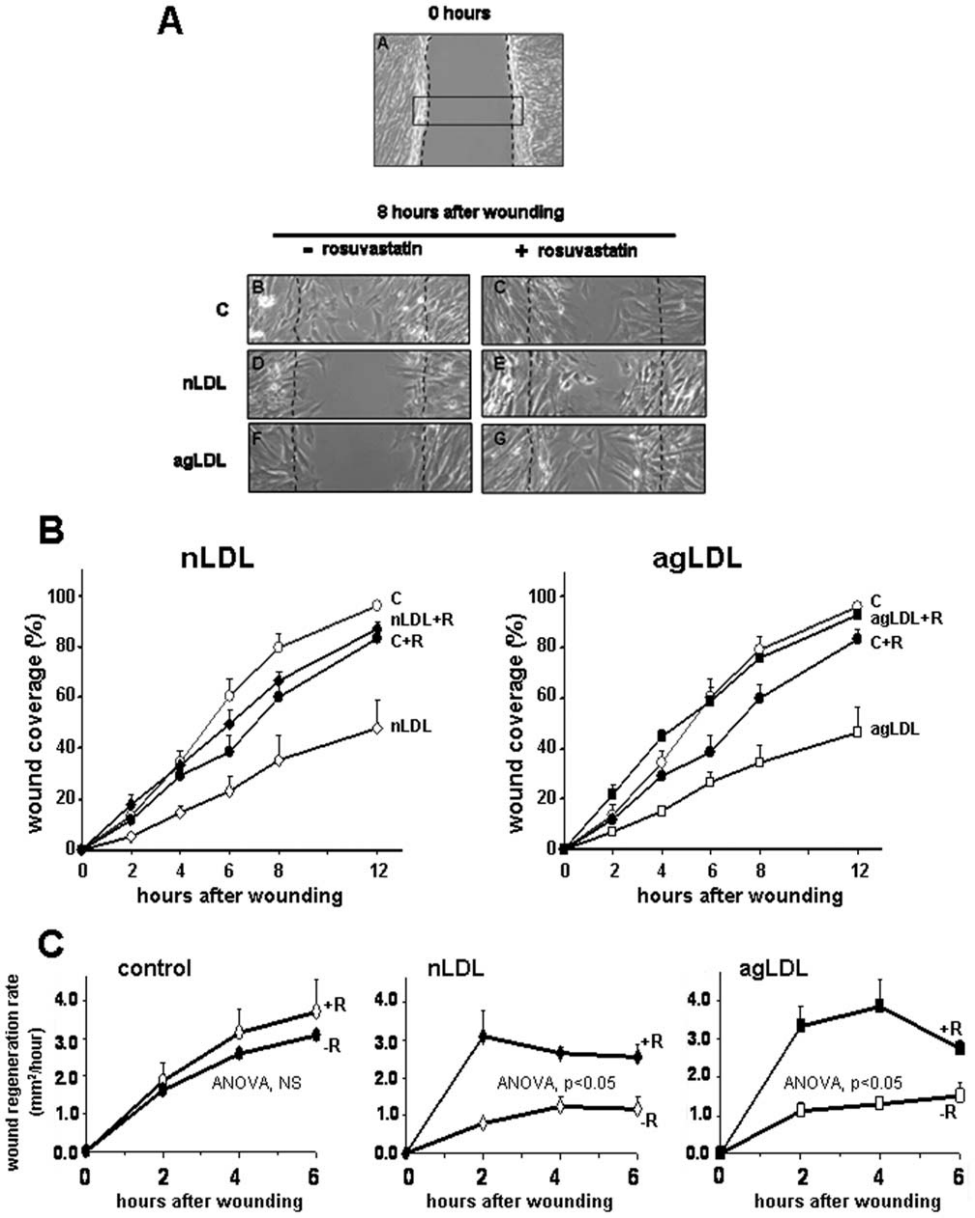
Rosuvastatin prevents the inhibitory effect of atherogenic LDL in wound repair by human VSMC. (**A**) Representative photomicrographs of VSMC treated without (B, D, F) or with (C, E, G) rosuvastatin in absence (B, C) or presence of nLDL (D, E), or agLDL (F, G) (**B**) Quantification of wound regenerated area (○: control; ⋄: nLDL-treated cells; ♦: nLDL+rosuvastatin; □: agLDL; ▪: agLDL+rosuvastatin). (**C**) Wound regeneration kinetics (mm^2^/hour) of control and LDL-groups treated without/with rosuvastatin (two-way ANOVA for rosuvastatin treatment and time). Results in B and C shown as mean±SEM (n=6 independent experiments).

**Figure 2:**
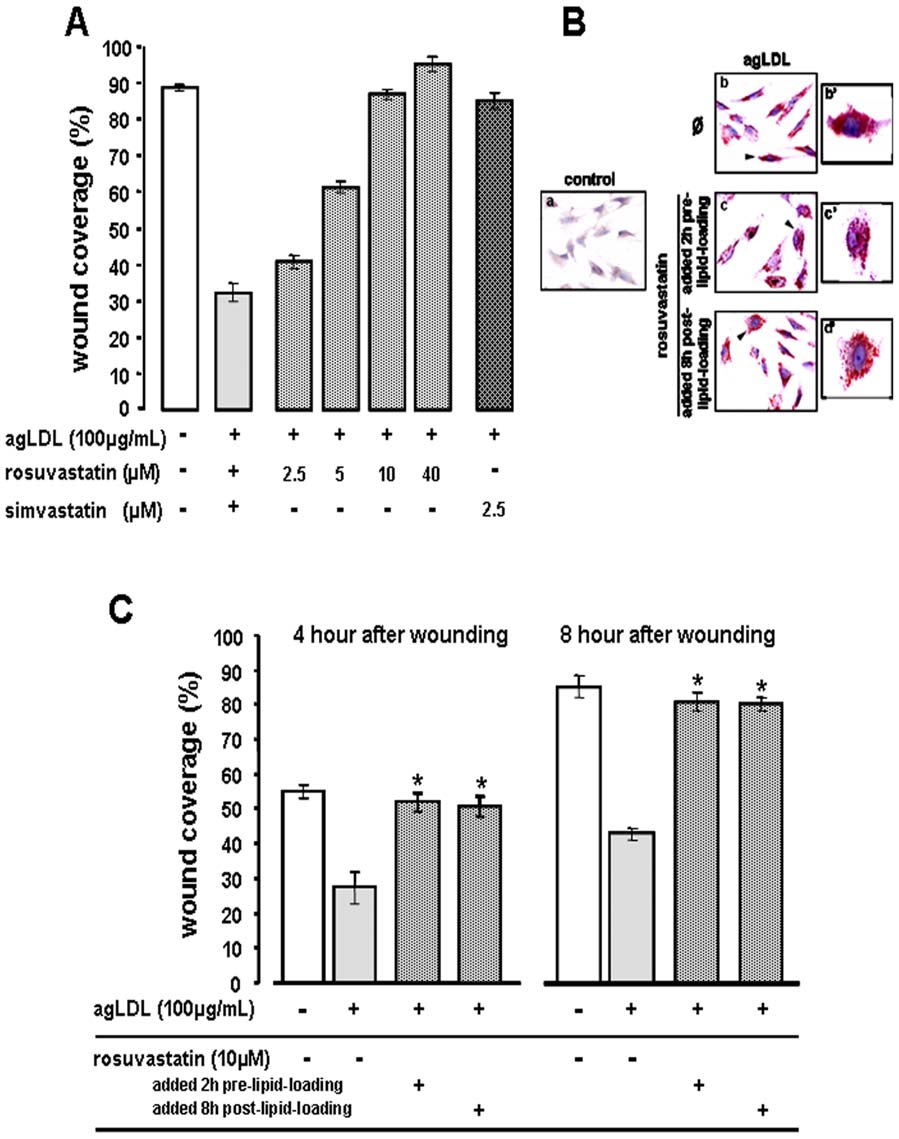
Dose-dependent response to rosuvastatin in cell migration of VSMC exposed to LDL. Bar diagram for (**A**) dose dependent effect of rosuvastatin (2.5–40 µM) in wound regeneration by VSMC treated without/with agLDL 8 hours after wounding. P<0.01 (Kruskal-Wallis test) for rosuvastatin dose-response effect and P<0.001 for simvastatin effect (Man-Whitney test) vs agLDL-group. (**B and C**) VSMC were treated without or with rosuvastatin (10 µM) since 2 hours before (2 h-pre LDL) or 8 hours after (8 h-post LDL) adding agLDL to the cell culture medium. **B:** staining with Herxheimer reagent (Sudan III and Sudan IV) (red colour) as a control for intra-cellular accumulation of lipids. Ø refers to agLDL-VSMC non treated with rosuvastatin. **C:** Results shown as mean±SEM (% of wounded area at time 0) refer % of the wound regenerated area 4 and 8 hours after wounding (n=4 independent experiments); * p<0.05 compared to the agLDL-group (Man-Whitney test).

Experimental studies using atherosclerotic animal models have suggested that the plaque-stabilizing effects of statins are related to an increase in VSMC and collagen content of the plaques [34]–[36]. Up to now, however, the mechanisms involved in statin-mediated enrichment and activity of VSMC in atherosclerotic plaques have not been fully characterized. In the present study, we investigated the mechanism by which rosuvastatin, a potent statin that has shown to induce stabilization of vascular lesions [31] and regression of clinical coronary atherosclerosis [27], rescues the VSMC migration kinetics and repair capacity that are impaired by accumulation of LDL.

**Figure 3:**
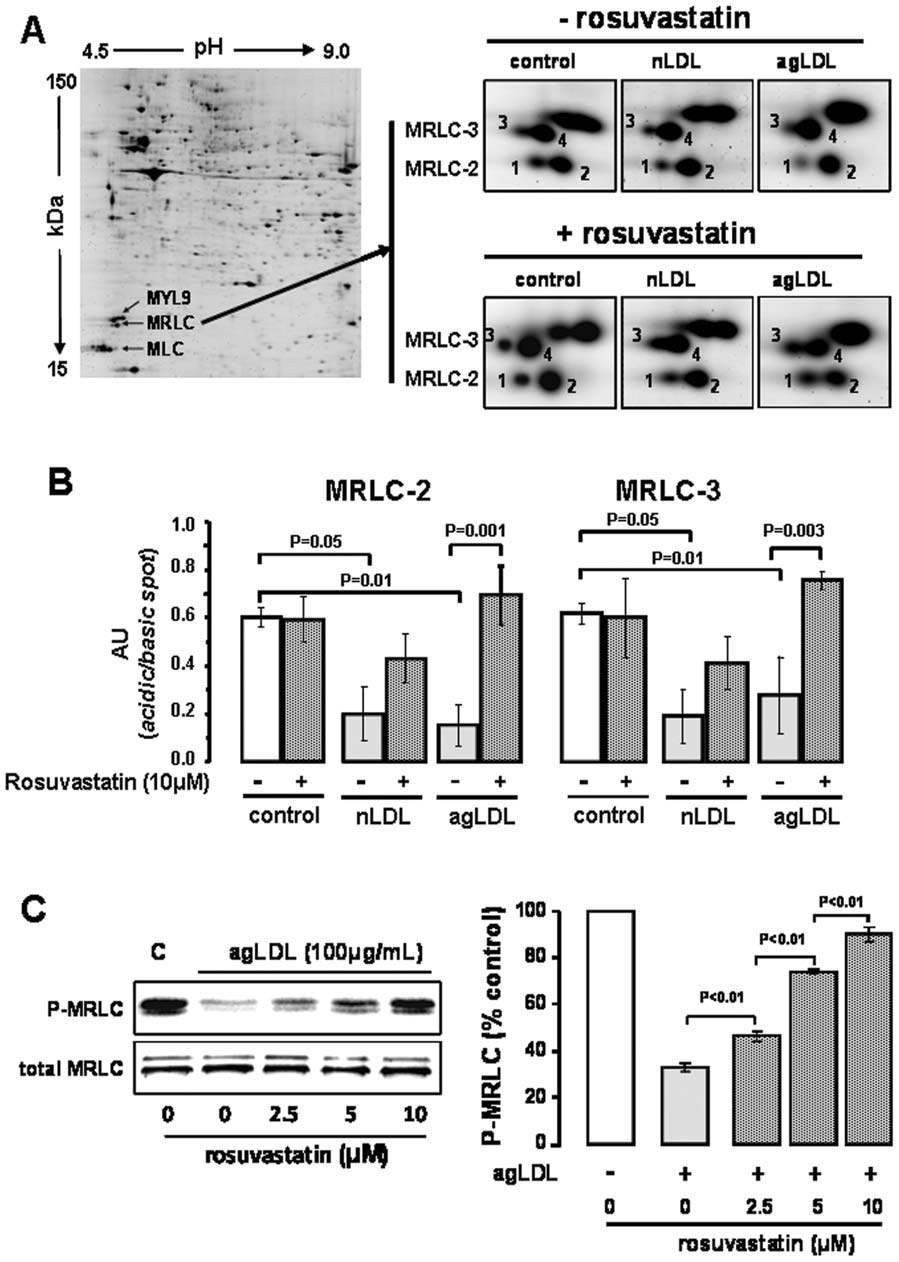
Rosuvastatin prevents MRLC dephosphorylation in VSMC exposed to LDL. (**A**) Representative 2-DE gel of the cytoskeleton/membrane fraction. Arrows show spots identified as MYL9 (sarcomeric-MRLC), non-sarcomeric-MRLC (MRLC) and alkali-MLC (MLC). Enlarged images for the proteomic pattern of the non-sarcomeric MRLC isoforms (MRLC-2, spot 1–2 and MRLC-3 spots 3–4). Note that numbers 1–2 and 3–4 denote double spots with different p*I* of the non-sarcomeric MRLC isoforms. (**B**) Bar-diagrams for ratios between the intensity of the spot with lower p*I* and the correspondent with higher p*I*. Results given as mean±SEM (n=3). P values for Man-Whitney test; only shown differences for P<0.05 (**C**) Western blot for phosporylated-MRLC (P-MRLC) and total MRLC in extracts of VSMC exposed to agLDL in the presence of different dose of rosuvastatin (2.5–10 µM). C denotes the control group. Bar diagram represents quantitative values (mean±SEM) of 3 independent experiments. P values for Man-Whitney test.

## Materials and Methods

### Human coronary vascular SMC culture, LDL preparation, and cell treatments

Primary VSMC were obtained by the explant technique from non-atherosclerotic coronary arteries of hearts, obtained from anonymized patients under heart transplantation surgery at the Hospital de la Santa Creu i Sant Pau, and cultured as described previously [37]. The study was approved by the Institutional Review Committee on Human studies of the Hospital de la Santa Creu i Sant Pau (Barcelona, Spain) that conforms to the Declaration of Helsinki and tissue samples were collected with written informed consent from the donors.

**Figure 4:**
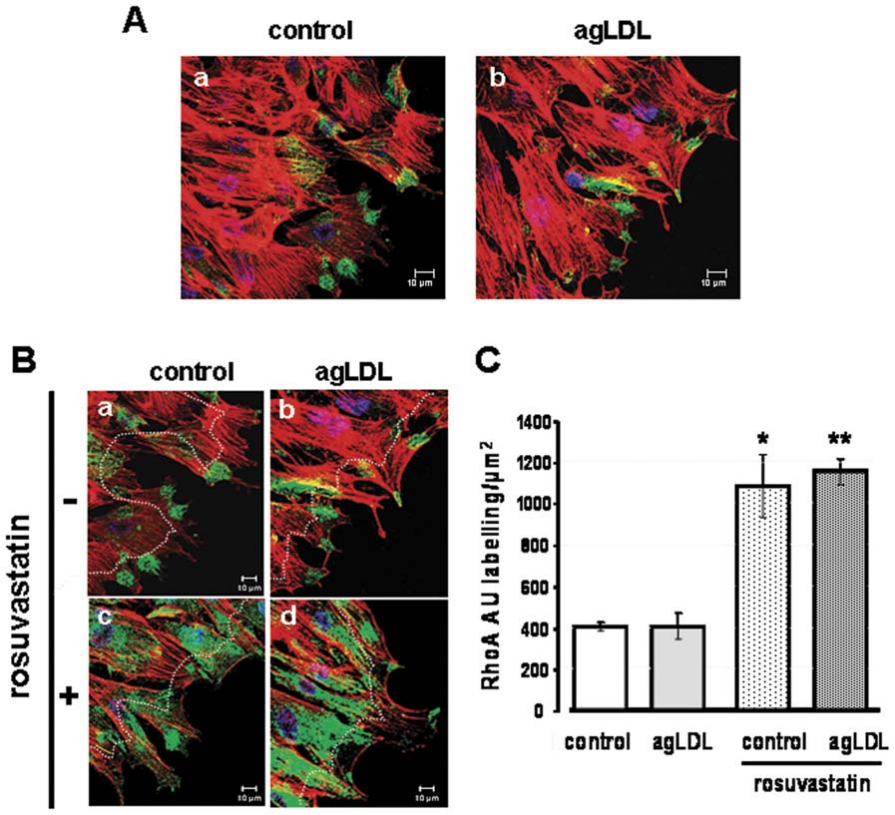
Rosuvastatin increases RhoA at the leading edge of migrating cells. Confocal microscopy for RhoA antigen labelling (green) in migrating cells (FCS-stimulated), 4 hours after the wound scratch. Immunocytology was performed in permeabilized cells. Red signal refers to F-actin (Alexa-Fluor 633 conjugated phalloidin). (**A**) Representative photomicrographs of RhoA labelling at the wound-border of control cells and agLDL-treated cells. (**B**) Effect of 10 µM rosuvastatin on RhoA localization at the leading-edge of control- and agLDL-VSMC. (**C**) Bar diagrams show quantitative levels (mean±SEM) of RhoA labelling over an area 15 µm wide, along the migrating edge (limited by dotted line in A). Intensity labelling signal was calculated using the LASAF Leica software and expressed in intensity/µm^2^ (results from 3 independent experiments). p<0.05 for (*) control+rosuvastatin *vs* control and (**) agLDL+rosuvastatin *vs* agLDL.

To investigate the effect of rosuvastatin on LDL-loaded VSMC, cell monolayers were preincubated with 10 µM rosuvastatin, concentration that corresponds to the blood level in patients treated with a high therapeutic-doses (40 mg), for 2 hours prior to the addition of 100 µg/mL native-LDL or aggregated-LDL (nLDL, agLDL) and incubated for further 16–24 hours. In a set of experiments, inhibitors of MLCK (ML9, Sigma), and ROCK (Y27632, Sigma) were added for the last 2 hours.

**Figure 5:**
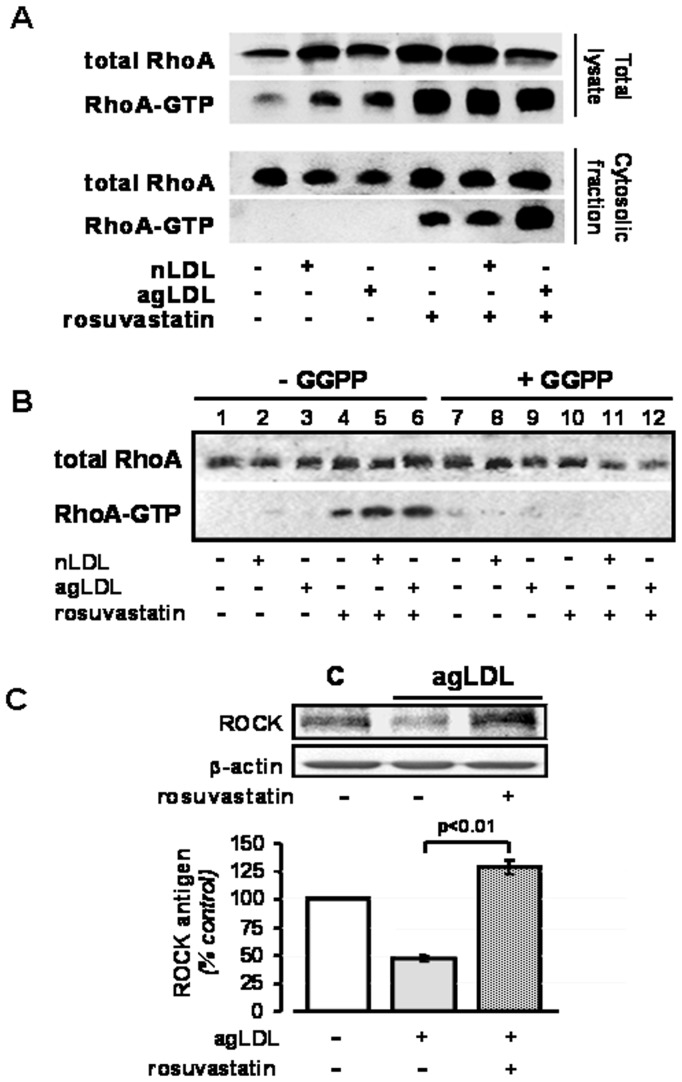
Increased levels of RhoA-GTP in cytosol of human VSMC exposed to agLDL in the presence of rosuvastatin. (**A**) Antigen (total RhoA) and active RhoA (RhoA-GTP) levels in total cell lysates and cytosol extracts of VSMC treated without/with rosuvastatin in absence/presence of nLDL and agLDL for 24 hours. Levels of active RhoA were determined by a pull down assay with GST-rothekin. Note the strong band for active RhoA in the cytosol of rosuvastatin treated cells. (**B**) Total and active RhoA levels in the cytosol fraction of VSMC treated without and with rosuvastatin +/−10 µM geranyl-geraniol pyrophosphate (GGPP), in the absence and presence of nLDL and agLDL for 24 hours. (**C**) Western blot analysis for ROCK antigen levels in cytosol extracts of cells exposed for 24 hours to agLDL in the absence and presence of rosuvastatin. C denotes the control non treated group.

For cell migration and immunohistochemical studies during VSMC wound repair, cells were grown on monolayers to confluence on glass bottom dishes (WPI, World Precision Instruments) and a linear wound was induced after the incubation period with/without rosuvastatin (+/− inhibitors), in the presence/absence of LDL. For proteomic and protein expression studies, treated cells were washed with ice-cold phosphate-buffered saline (PBS) and harvested in PBS containing 5 mM EDTA and stored at −80°C, until used.

**Figure 6:**
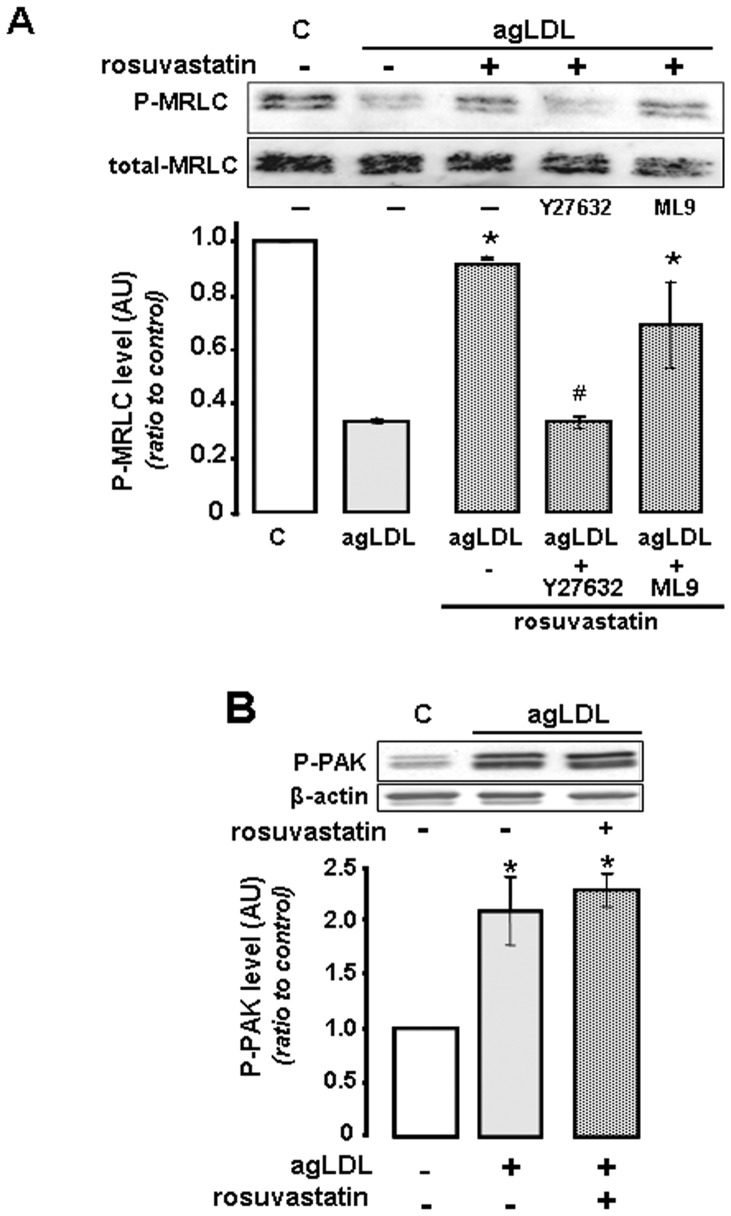
Rosuvastatin induced MRLC-phosphorylation in agLDL-VSMC is dependent on the activity of the RhoA/ROCK signaling pathway. VSMC incubated without/with agLDL, in the absence/presence of rosuvastatin analyzed by western blot for (**A**) P-MRLC in agLDL-VSMC incubated with rosuvastatin in the absence/presence of inhibitors for MLC-Phosphatase (Y27632) and MLCK (ML9). Bar diagram expresses mean±SEM of 3 independent experiments. *P<0.05 (Mann Whitney test) vs agLDL. # P<0.05 (Mann Whitney test) *vs* agLDL+rosuvastatin group. (**B**) phosphorylated PAK (p21-activated kinase) levels in VSMC exposed to agLDL in the absence/presence of rosuvastatin. Bar diagram represent the mean±SEM of 3 independent experiments. P<0.05 (Mann Whitney test), * *vs* control group.

Human LDL (density 1.019 to 1.063 g/mL) were prepared by ultracentrifugation from pooled sera of normocholesterolemic volunteers. agLDL were generated by vortexing LDL (1 mg/mL), according to the method previously described by Guyton et al [38]. As largely demonstrated, the vortexing-generated aggregated LDL (agLDLs) share structural characteristics, size, and electrophoretic mobility with the high atherogenic LDL aggregates retained in atherosclerotic arterial intima due to versican fusion [14], [38]–[40]. Intracellular lipid-accumulation in VSMC was regularly controlled by cell-staining with Herxheimer reagent (Sudan III and Sudan IV). In all experiments LDL-oxidation (before and after aggregation) was excluded by measuring thiobarbituric acid-reactive substances and by a monoclonal antibody 4E6-based ELISA (Mercodia) [18].

**Figure 7:**
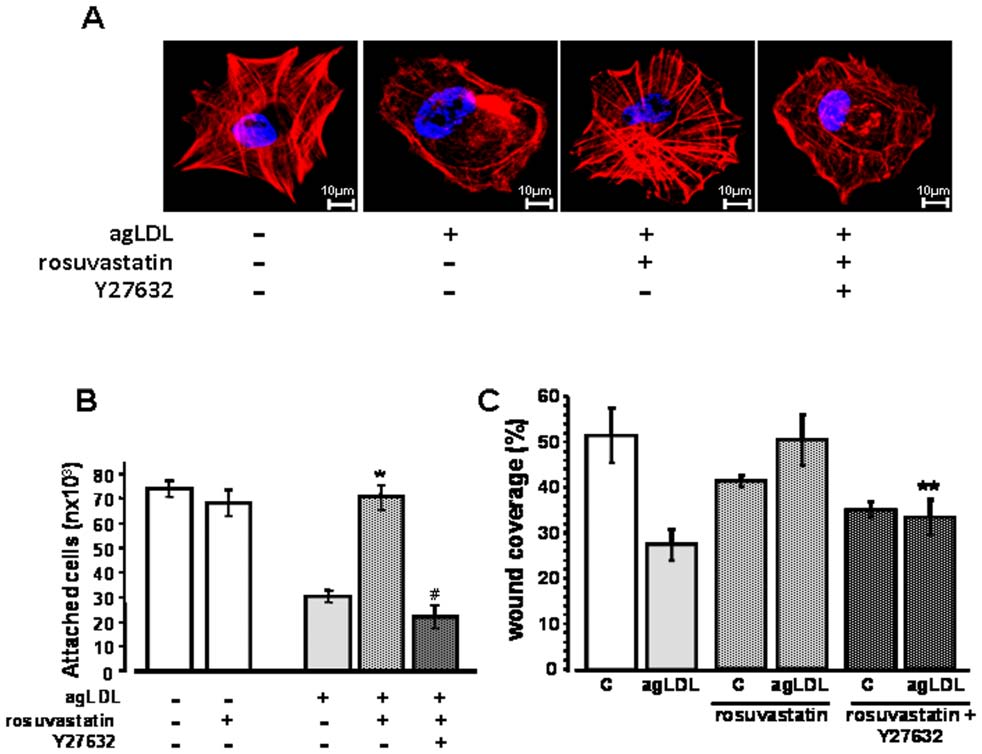
Rosuvastatin induced actin-cytoskeleton reorganization, cell adhesion and migration in agLDL-VSMC is dependent on the RhoA/ROCK signaling pathway. agLDL-VSMC incubated with rosuvastatin in absence/presence of inhibitors for ROCK (Y27632) or MLCK (ML9) were analyzed for (**A**) filamentous actin organization during cell attachment. Note that rosuvastatin-promoted filamentous actin organization in LDL-VSMC was prevented by Y27632 (**B**) cell attachment (expressed as percentage of the control group) (*) *vs* agLDL and (#) *vs* agLDL+ rosuvastatin group, p<0.01 (Mann Whitney test). (**C**) wound repairing (*) *vs* agLDL p<0.01 (Mann Whitney test) and (#) *vs* agLDL+ rosuvastatin group, p<0.01 (Mann Whitney test). In B and C, bar diagrams represent the mean±SEM of 3 independent experiments.

### Cell migration and wound repair assay

A double sided scrape-wound was made on confluent VSMC monolayers, as previously described [18]. Cell migration and wound repair were analyzed over a period of 12 hours (37°C) on 10% FCS-stimulated cells. Images were taken under a ×10 lens, using an inverted microscope (Leica DMIRE2) attached to a video SPOT camera (Leica-DFC350FX), on 3 different fields along the linear scratch covering more than the 95% of the wound. The area free of cells in each field was calculated using ImageJ software, and values averaged. Measurements were made at time 0 and during cell migration (2, 4, 8 and 12 hours). Cell migration and wound repair were indirectly assessed by measuring the residual cell-denuded area. Values were expressed as a percentage of the cell-depleted area at time 0.

**Figure 8:**
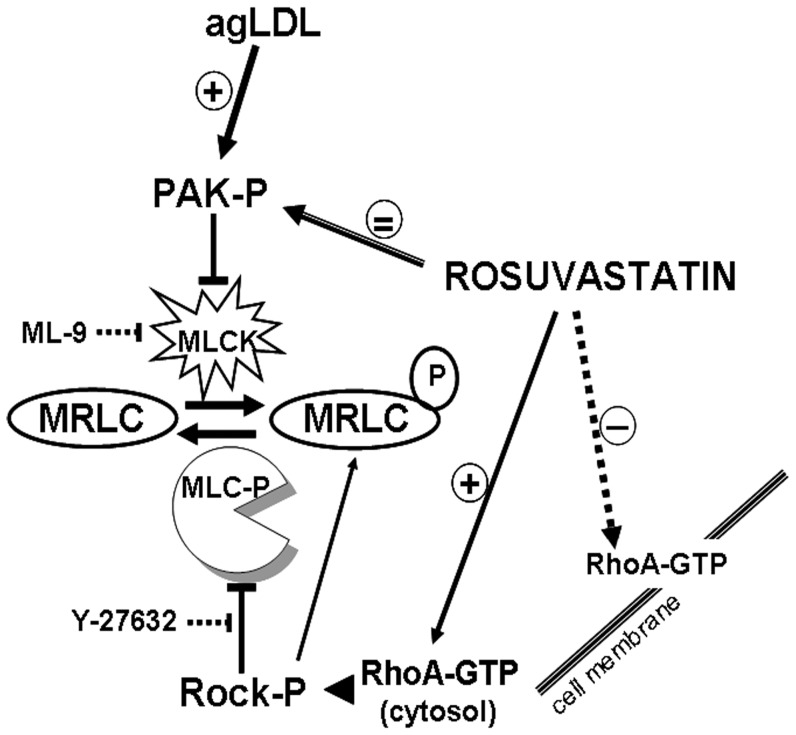
Scheme for signaling pathways regulating MRLC-phosphorylation in human VSMC. Potential mechanisms involving agLDL and rosuvastatin effects are indicated. *MRLC: Myosin regulatory light chain; MLCK: Myosin light chain kinase. MLC-P: Myosin light chain phosphatase; ROCK: Rho-associated protein kinase; PAK: p21-activated protein kinase. RhoA: Small G proteins; agLDL: aggregated low density lipoproteins.*

### Cell Proliferation

Arrested VSMC were incubated for 24 hours with 10 µM rosuvastatin and 100 µg/mL nLDL or agLDL in medium containing 0.5 µCi/mL of [3H]thymidine (Amersham) and cell proliferation was determined as previously described [41].

### Cell adhesion assay

Subconfluent VSMC, treated with/without rosuvastatin in the presence/absence of agLDL (16 hours), were harvested and resuspended in 5% FCS-M199 medium. Afterwards, 1×10^5^ cells were seeded on FCS-coated glass bottom dishes and further incubated for 3 hours in the presence/absence agLDL, without/with rosuvastatin +/− Y27632. After 3 hours, the attached cells were fixed with 4% paraformaldehyde for confocal microscopy studies or released by trypsination, stained with trypan blue for determination of cell viability and counted in a Neubauer-chamber. Experiments were performed in replicates and mean values were calculated.

### Protein extraction and proteomic analysis

Frozen cell pellets were sequentially extracted in tris- and urea buffers to separate the cytosolic and membrane/cytoskeleton-enriched protein fractions [18]. Total cell lysates were made in RIPA-buffer. (50 mM Tris HCl pH 8; 150 mM NaCl; 1% NP-40; 0.5% sodium Deoxycholate; 0.1% SDS). Protein concentration was measured with 2D-Quant Kit (GE-Healthcare), as indicated by the manufacturer.

For the proteomic analysis, sample contaminants were removed (2D-CleanUp Kit, Amersham) and proteins (120 µg) separated by 2-dimensional electrophoresis (2-DE) as previously described [18]–[19]. Protein spots in the gels were labelled by fluorescence (Flamingo labelling, BioRad), scanned (Typhoon, GE-Healthcare), and analysed for differences in the protein patterns between groups with PD-Quest 8.0.1 software (8.0.1, Bio-Rad) [18]–[19].

Protein identification was performed by matrix-assisted laser desorption/ionisation-time of flight (MALDI-ToF) mass spectrometry (Ettan MALDI-ToF Pro, GE Healthcare) as previously described by our group [18].

### Western blot analysis

Protein antigen levels in cytosol and membrane fractions were analyzed by western blot, as described previously [18]–[19] using following primary antibodies: monoclonal anti-MRLC (Abcam, dilution:1/500), polyclonal anti-phosphorylated-MRLC (Abcam, dilution:1/500), monoclonal anti-RhoA (Santa Cruz, diution:1/500), polyclonal anti-p160ROCK (Santa Cruz, dil:1/500), monoclonal anti-phosphorylated-PAK1 (Abcam, dilution:1/10000), and monoclonal anti-β-actin (Abcam, dilution:1/5000). Western blot bands were detected by chemiluminescence using a peroxidase enzymatic reaction (Supersignal, Pierce) and quantified with a ChemiDoc™ XRS system using the Quantity-One 1-D analysis software (Bio-Rad).

### Immunofluorescence and confocal microscopy

Cells were fixed with 4% paraformaldehyde, permeabilized (0.5% Tween-PBS), and blocked with 1% bovine serum albumin (BSA). P-MRLC was detected with a rabbit polyclonal antibody (Santa Cruz) and RhoA with a monoclonal-antibody (Santa Cruz). The secondary antibodies were Alexa Fluor-488 conjugated anti-rabbit or anti-mouse antibodies (Molecular Probes dilution 1/100). Negative controls of primary and secondary antibody staining were run with each set of experiments. F-actin was stained with Alexa Fluor-633 phalloidin (Molecular Probe) and nuclear counterstain with Hoechst (Molecular Probes). Labelled cells were examined in a Leica inverted fluorescence confocal microscope (Leica TCS SP2-AOBS). Fluorescent images were acquired in a scan format of 1024×1024 pixels at intervals of 0,1 µm (20 slides) and processed with the TCS-AOBS software (Leica). Maximal intensity projection values were calculated using the LASAF Leica Software and given as AU/µm^2^.

### RhoA Activity Assay

Levels of active RhoA were measured in cytosol extracts and in total cell lysate with a RhoA activation assay kit (BK036, Cytoskeleton) based in a pull-down technique, following the manufacturer's instructions. Briefly, VSMC were starved 24 hours and treated as usual before harvesting, either with Tris 40 mM (cytosol extract) or with lysis buffer provided by the RhoA activation assay kit. Protein concentration was determined immediately (Precision Red Advanced Protein Assay Reagent, ADV02, Cytoskeleton) and equal amount of extracts were incubated with GST-Rhotekin sepharose beads in order to pull down active RhoA. Pulled down beads were washed twice and resuspended in Laemmli sample buffer. The complete pulled down sample, containing only active RhoA, was analyzed by western blot, as described above.

### Statistical analysis

Results are presented as mean±SEM and the number of experiments is shown in every case (see figure legends). Statistical differences between groups were analyzed by parametric tests as T-Student, one-way analysis of variance (ANOVA) or two-way ANOVA, followed by the Fisher's PLSD test for group differences or by non-parametric tests (Kruskal Wallis or Mann-Whitney), as indicated. P value of 0.05 or less was considered significant.

## Results

### Rosuvastatin prevents LDL-induced impairment of VSMC-migration

Our previous studies have shown that agLDL hamper mobility of human coronary VSMC [18]–[19]. Here, treatment of VSMC with 10 µM rosuvastatin prevented LDL-induced impairment in migration kinetics and repaired the cell-depleted areas (Fig. 1A). Differences in cell migration due to treatment with rosuvastatin were already observed 2 hour after wounding (Fig. 1B, Table S1). Indeed, wound repair kinetics (mm^2^/hour) were significantly higher in cells treated with rosuvastatin than in cells incubated with LDL alone (P<0.01 for both nLDL and agLDL). In contrast, rosuvastatin did not affect wound repair kinetics in the control non-LDL treated group (Fig. 1C; P=NS for control *vs* control+rosuvastatin group).

As shown in Fig. 2A, the effect of rosuvastatin on migration of VSMC exposed to LDL (LDL-VSMC) was dose-dependent (2.5 to 10 µM). Thus, rosuvastatin at 10 µM, concentration that corresponds to the blood concentration in patients treated with high therapeutic-doses (40 mg), restored to control levels the VSMC migration that was interfered by LDL. A non-statistically significant increase with respect to 10 µM was found with 40 µM rosuvastatin, concentration above the therapeutic range. Simvastatin elicited a similar effect at a lower concentration according to its lipophilic structure. The effect of rosuvastatin in restoring VSMC migration kinetics was already evident at 4 hours. Additionally, in a set of experiments, VSMC were lipid-loaded (100 µg/mL agLDL for 8 hours [40], Fig. 2B) and then rosuvastatin (10 µM) added for an additional 8 hour-period before wounding. Rosuvastatin restored the migration capacity of VSMC to similar level when treating both lipid-loaded and lipid-unloaded cells (Fig. 2C).

The effects of rosuvastatin on the recovery of wound repair function in lipid-loaded-VSMC were not caused by cell proliferation. Indeed, a cell [3H]thymidine incorporation assay demonstrated, as expected, that rosuvastatin (10 µM) significantly reduced (about 80%) proliferation in lipid-loaded VSMC (Table S2).

### Rosuvastatin reverts the changes induced by LDL on the proteome of MRLC in human coronary VSMC

2-DE and MALDI-TOF techniques were used to compare the proteomic pattern of the non sarcomeric-MRLC isoforms MRLC-2 (Swiss-Prot number: O14950; observed p*I* 5.1, observed MW 21.5 kDa) and MRLC-3 (Swiss-Prot number: P19105; observed p*I* 5.0, observed MW 22.2 kDa) in human VSMC treated with/without rosuvastatin during exposure to native/aggregated LDL (Fig. 3).

MRLC isoforms appeared as a double spot of different p*I* (MRLC-2: spots 1,2; MRLC-3: spots 3,4; Fig. 3A), which were related to different phosphorylation states. For each MRLC-isoform, the ratio between the intensity level of the spot with lower p*I* (more phosphorylated forms) and this with higher p*I* (less phosphorylated forms) was calculated. Treatment of VSMC with rosuvastatin reverted dephosphorylation induced by LDL in MRLC-3 and MRLC-2. The effect was markedly evident for the agLDL-treated group (Fig. 3B).

Western blot analysis of agLDL-VSMC treated with different rosuvastatin concentrations revealed a progressive increase in P-MRLC compared to untreated cells (Fig. 3C), being the increase already evident with the lowest concentration of rosuvastatin (2.5 µM). The highest concentration of rosuvastatin (10 µM) increased levels of P-MRLC to 90% of the control group (without agLDL).

### Rosuvastatin increases P-MRLC levels at the migration front of agLDL-treated VSMC

We have also examined the effect of rosuvastatin on the subcellular distribution of P-MRLC by confocal microscopy (Fig. S1). Migrating agLDL-VSMC treated with rosuvastatin shows a P-MRLC labelling signal intensity similar to the control group. Contrarily, the P-MRLC labelling that is very weak at the border of the wounded area in agLDL treated VSMC, shows a significant increase in presence of rosuvastatin. Rosuvastatin did not show any effect on the P-MRLC in control VSMC migrating into the wounded area.

### Effect of rosuvastatin on the subcellular distribution of RhoA in human VSMC

Because HMG-CoA reductase inhibitors have shown to regulate isoprenylation of small GTP-binding proteins, we analyzed subcellular RhoA levels in cytosol, cytoskeleton-associated and membrane fractions in the lipid loaded VSMC (Fig. S2). Rosuvastatin treatment significantly increased RhoA protein levels in the cytosol of the VSMC (>1.5–2.0fold, *p*<0.05), while weakly decreased RhoA in the cytoskeleton associated fraction. RhoA levels were markedly lower in the membrane fraction of rosuvastatin treated cells (>8fold decrease, p<0.01).

By confocal microscopy analysis, migrating VSMC consistently showed high RhoA levels at the leading edge of the cells, either in the presence or absence of agLDL (Fig. 4A). Rosuvastatin significantly increased RhoA labelling at the migrating front edge (compare Fig. 4B-a and 4B-b with 4B-c and 4A-d). Taking as reference the bulk of RhoA labelling in the control group (Figure 4A), we have defined a 15 µm wide area along the border of the wound (boundary marked by dot lines in Fig. 4B) to quantify the effect of rosuvastatin on the RhoA-level in the migrating VSMC. As shown in Fig. 4C, rosuvastatin induced >2.5fold increase of RhoA either in control and LDL-treated cells (P<0.05 both for control and agLDL-VSMC).

### Effect of rosuvastatin on RhoA-GTP levels

Levels of RhoA-GTP (active RhoA) were analyzed by a pull down assay with GST-rothekin in total cell lysates and cytosol extracts (Fig. 5A). RhoA-GTP was detected in the total cell lysates of both control and LDL-treated VSMC, but never in the cytosolic fraction of these cells. Levels of RhoA-GTP were significantly increased when cells were treated with rosuvastatin. RhoA-GTP levels increased both in total cell lysates and in the cell cytosol-fraction (Fig. 5A). There was no increase in RhoA-GTP in the cytosol of cells coincubated with rosuvastatin and 10 µM geranyl-geraniol pyrophosphate (GGPP) (Fig. 5B). Besides, the level of cytosolic ROCK, the major downstream target of active RhoA, was 2.5fold increased (P<0.01) in agLDL-VSMC treated rosuvastatin (Fig. 5C).

By 2-DE we identified the p50RhoGAP (Q07960, p*I* 5.9, MW 50 kDa), a member of the GTPase activating protein family that catalyzes inactivation of RhoA. This protein was significantly overexpressed by agLDL-loading of VSMC (mean±SE intensity level in control *vs* agLDL group: 0.28±0.01 *vs* 0.41±0.05 AU, P=0.03, Fig. S3). Treatment with rosuvastatin decreased p50RhoGAP in agLDL-VSMC to levels detected in control cells (agLDL+rosuvastatin: 0.27±0.01 AU, P=0.02 *vs* agLDL).

### Rosuvastatin-induced MRLC-phosphorylation, cytoskeleton reorganization, cell adhesion and migration in agLDL-VSMC is dependent on ROCK-signaling

We next explored whether the RhoA-downstream pathway was involved in the effect of rosuvastatin on the cytoskeleton dynamics in agLDL-VSMC. MRLC-phosphorylation is mainly influenced by the opposing activities of MLC-kinase (MLCK) and MLC-phosphatase (MLC-P), which in turn are regulated by p21-activated protein kinase (PAK-1) and Rho-dependent kinase (ROCK) [42]–[43].

As shown in Fig. 6A, the specific ROCK inhibitor Y27632 (10 µM) abolished the increase in P-MRLC induced by rosuvastatin in agLDL-VSMC (phosphorylation ratio agLDL: 0.34±0.00; agLDL+rosuvastatin: 0.92±0.01, agLDL+rosuvastatin+Y27632: 0.33±0.04; P<0.01). Addition of the MLC-kinase inhibitor ML-9 induced a non-significant reduction of P-MRLC (agLDL+rosuvastatin+ML9: 0.70±0.16; P=NS vs agLDL+rosuvastatin). The levels of phosphorylated-PAK1 (P-PAK1) were 2fold increased (p<0.05) in agLDL- VSMC with respect to control cells, an effect not reversed by rosuvastatin (10 µM) (Fig. 6B).

In a subset of experiments, VSMC were incubated with agLDL and treated with/without rosuvastatin for 16 hours, and then were seeded and allowed to attach on glass plates for 3 hours. Confocal analysis of actin fibres labelled with Alexa Fluor-633 phalloidin (Fig. 7A) demonstrated that rosuvastatin strongly promoted filamentous actin organization in LDL-treated VSMC, whereas co-incubation with 10 µM Y27632 prevented the effect of rosuvastatin. Three hours after seeding, 73±3% of the plated agLDL-VSMC were attached in the presence of rosuvastatin whereas only 30±4% in the absence of rosuvastatin (P<0.01) (Fig. 7B). Co-incubation of rosuvastatin with 10 µM Y27632 decreased cell attachment to the level of the agLDL-VSMC (22±10%; P<0.01 vs LDL+rosuvastatin group; P=NS vs LDL-group).

The ROCK inhibitor Y27632 consistently reduced the reversing effect of rosuvastatin on LDL-induced impairment in cell migration (Fig. 7C). Thus, when agLDL treated cells were co-incubated with Y27362 and rosuvastatin the percentage of wound coverage did not differ significantly from the basal levels observed in the absence of rosuvastatin. In addition, Y27362 retarded the induction of migration (2 hours) during an in vitro wound healing assay in control VSMC, but no significant difference was seen at longer times. Y27362 had also no effect when VSMC were treated only with rosuvastatin.

## Discussion

Atherosclerotic plaques with large lipid cores and paucity of VSMC have an increased risk of rupture. We have recently demonstrated that LDL impair VSMC migration kinetics and induce changes in cytoskeleton proteins [18]–[19], mechanisms by which LDL could contribute to the relative loss of human VSMC in vulnerable plaques. The role of infiltrated LDL in vascular remodelling and repair is a key issue to understand progression of atherosclerosis. Modified LDL are found within the diseased vessel in close contact with smooth muscle cells. These infiltrated LDL aggregate and accumulate by binding to extracellular matrix proteoglycans [9], [11], [13] becoming targets for oxidation, glycation, and other enzymatic modifications. Therefore, aggregation is the initial modification suffered by infiltrated LDL affecting VSMC function and phenotype. Indeed, in addition to cytoeskeleton protein modifications, LDL downregulate expression of LDLR whereas upregulate expression of LRP1, a receptor for agLDL in VSMC [14]–[15]. Thus, LRP1 by internalizing significant amounts of cholesteryl esters from agLDL contributes to the transformation of VSMC into “foam cell-like” cells [15]–[17]. Overexpression of LRP1 has been found in atherosclerotic plaques in animal models and in human plaques of increasing severity [44]–[46].

HMG-CoA reductase inhibitors have shown to reduce clinical cardiovascular disease presentation [23]–[24]. Among them, rosuvastatin, the latest introduced, has shown efficacy in primary presentation [26] and regression or halt of disease burden [27], [47]. Several studies have shown that statins favourably alter the fibromuscular composition of the plaques both in hyperlipidaemic ApoE^−/−^ mice with increased collagen content and increased SMC-α-actin [34]–[35] and human carotid plaques [33].

In this study, we aimed at investigating the mechanisms by which rosuvastatin exert plaque remodelling effects and report that rosuvastatin elicits a counteracting regulation of the impairing effects of LDL on VSMC function and migration kinetics.

Here, we report for the first time that rosuvastatin prevents in a dose-dependent manner the agLDL-induced dephosphorylation of MRLC and the agLDL-detrimental effects on actin fiber formation. Additionally, rosuvastatin affects intracellular localization of phosphorylated-MRLC that is found concentrated at the front edge of the migrating cells in the treated VSMC. Phosphorylated-MRLC activates myosin II through its ATPase motor domain, a critical regulator of cytokinesis and as consequence of cell migration [8], [29].

Rho GTPases are key regulators of cytoskeleton dynamics in a wide variety of morphogenetic events, including cell migration [48]–[49]. Earlier studies in migrating cells had linked Rac1 to the protrusion of the leading edge and RhoA to the contractility control at the back of the cell [50]. However, as shown here recently RhoA has been reported to accumulate at the front of migrating cells. To this respect, a Rab13 dependent trafficking of RhoA from the junctions to the leading edges has been shown in migrating endothelial cells [51]. Besides, active RhoA has been detected in lamellipodia and filopodia of transendothelial migrating T cells where it was involved via mDIA or ROCK signalling in regulation of actin-myosin-mediated protrusion and retraction events at the leading edge [52]. Additionally, a spatio-temporal dynamic process coordinated with other Rho GTPases as Rac and Cdc42 has been also reported [49].

HMG-CoA reductase inhibitors block the synthesis of farnesyl pyrophosphate (FPPP) and geranylgeranyl pyrophosphate (GGPP) isoprenoids and thereby disrupt posttranslational isoprenylation and intracellular translocation from cytoplasm to membrane of isoprenylated proteins involved in cell signalling, such as the members of the Rho family [53]. We have recently demonstrated that simvastatin abrogates agLDL-induced translocation of RhoA to cell membrane in VSMC [54]. Here, we further report that rosuvastatin leads to a highly significant increase in functional GTP-RhoA in the cytosol of VSMC, along with a several-fold decrease in the amount of RhoA partitioning in the membrane compartment. These new findings in VSMC are consistent with those of previous studies in monocytes, HEL cells and endothelial cells showing that statins promote Rho-GTP binding and activation [55]–[56]. Besides, we report a significant increase of RhoA protein at the front edge of migrating LDL-loaded-VSMC in response to rosuvastatin. All together, our results suggest that anchoring of active RhoA in the cell membrane is not required for the increase in cell motility induced by rosuvastatin in LDL-loaded cells. The functional relevance of this finding is underscored by studies showing that unprenylated G-proteins are also biologically active [55], [57]–[58].

Further studies are needed to determine the mechanism underlying rosuvastatin-mediated cytosolic RhoA activation. One potential explanation is that HMG-CoA reductase inhibitors disrupt the interaction of small G-proteins with its negative regulator RhoGDI (Rho guanine nucleotide dissociation inhibitor) [56], as they do with GTPase-activating proteins (GAPs) that accelerate GTP hydrolysis. These interactions depend on protein isoprenylation [59]. Interestingly, in our study, supplementation of VSMC cultures with GGPP prevented rosuvastatin-induced RhoA-GTP increase in the cellular cytosol. Beside, we observed higher p51GAP levels in LDL-loaded-VSMC in the absence than in the presence of rosuvastatin.

Functional RhoA plays a key role in regulating molecular responses linked to actin dynamics [60]. RhoA-GTP by binding to the Rho-binding domain (RBD) of ROCK disrupts the interaction between the catalytic N-terminal and the inhibitory C-terminal regions of the enzyme, stimulating in turn the phosphotransferase activity of ROCK [61]. Thus, RhoA regulates MRLC-phosphorylation through its downstream effector ROCK and through MLC-phosphatase. In addition, ROCK may also phosphorylate Ser19 in MRLC, the same residue that is phosphorylated by MLC kinase (MLCK) [43]. In LDL-loaded-VSMC, ROCK inhibition completely reverted rosuvastatin induced MRLC-phosphorylation, whereas MLC-kinase (MLCK) inhibition did not induced a significant effect. Taken together, these results indicate that rosuvastatin induces MRLC-phosphorylation, enhances cytoskeleton organization and promotes cell adhesion and migration of LDL-loaded-VSMC through a mechanism involving activation of the RhoA/ROCK downstream pathway.

MLCK activity is inhibited by phosphorylated p21-activated kinase (P-PAK) [42]. Rosuvastatin did not revert the increase in phosphorylated PAK induced by agLDL, thus emphasizing the relevance of the ROCK signalling pathway in MRLC-phosphorylation. Thus, we propose that agLDL induced MRLC-dephosphorylation in VSMC is linked to high levels of P-PAK and to a low MLCK-activity, whereas rosuvastatin counteracts the agLDL effect on P-MRLC by a mechanism that mainly depends on the increase of cytosolic RhoA-GTP and its target ROCK, which in turn blocks MLC-phophatase activity and activates MRLC-phosphorylation (Fig. 8). In control cells, without LDL, there is a redundancy in the mechanisms involved in MRLC-phosphorylation that overcome the effect of rosuvastatin during cell migration. Indeed, rosuvastatin, in the absence of LDL, does not induce migration in VSMC, in agreement with the inhibitory effect in motility attributed by others to rosuvastatin in absence of LDL [62]–[63]. Moreover, rosuvastatin might affect VSMC migration through additional mechanisms directly involving activation/inhibition of other small G-proteins, which will require future studies.

In summary, current imaging methods in clinical studies [27] have shown that rosuvastatin reduces atherosclerotic plaque burden and the vulnerability of the plaques. Here, we demonstrate that these effects of rosuvastatin could be mediated by counteracting the impairing effects of LDL in MRLC phosphorylation and cell migration/repair function of lipid-loaded-VSMC. These mechanisms may strongly contribute to stabilize lipid-rich atherosclerotic plaques.

## Supporting Information

Table S1 **Effect of rosuvastatin treatment on regeneration of cell depleted areas (% of wounded area at time 0) by coronary derived human VSMC at different time periods after wounding.**
(DOC)

Table S2 **Effect of rosuvastatin (10 µM) on levels of [3H]thymidine incorporation in human VSMC exposed to 100 µg/mL nLDL or agLDL.**
(DOC)

Figure S1 **Effect of rosuvastatin on subcellular localization of phosphorylated MRLC in migrating VSMC during wound healing.** Confocal microscopy of migrating cells (10%FCS-stimulated), 4 hours after wounding. Human coronary VSMC treated as shown for 16 hours. Cells were labelled for P-MRLC (Alexa Fluor 488-conjugated, signal in green) and F-actin (Alexa-Fluor 633 conjugated phalloidin, signal in red).
(TIF)

Figure S2 **Increase of RhoA levels upon rosuvastatin treatment in the cytosol of human VSMC.** RhoA and β-actin in cytosol, cytoskeleton-membrane and membrane extracts of VSMC with/without LDL and treatment with rosuvastatin or vehicle for 24 hours. The histograms show results expressed as % of the controls after normalization for β-actin. Results are given as mean±SEM of 3 independent experiments.
(TIF)

Figure S3 **Rosuvastatin decreases p50RhoGAP level in LDL-treated VSMC.** Representative 2-DE gel of the urea/detergent-soluble fraction of human VSMC. Enlarged images correspond to the gel area where p50RhoGAP (pI 5.9, MW 50 kDa) was detected. Rosuvastatin markedly decreases the labelling signal for p50RhoGAP (agLDL+rosuvastatin) in VSMC treated with agLDL.
(TIF)
