# Supplementary material for: LDL-Induced Impairment of Human Vascular Smooth Muscle Cells Repair Function Is Reversed by HMG-CoA Reductase Inhibition
Source: PLoS One. 2012 Jun 12;7(6):e38935. doi: 10.1371/journal.pone.0038935 (PMC3373563; doi:10.1371/journal.pone.0038935)
Supplement: Table S1 — Effect of rosuvastatin treatment on regeneration of cell depleted areas (% of wounded area at time 0) by coronary derived human VSMC at different time periods after wounding. (DOC) [file pone.0038935.s001.doc]

**Table S1**. Effect of rosuvastatin treatment on regeneration of cell depleted areas (% of wounded area at time 0) by coronary derived human VSMC at different time periods after wounding.

| **Treatment** | **- Rosuvastatin** | **+ Rosuvastatin** | **P value*** |
| --- | --- | --- | --- |
| 2 hours |  |  |  |
| control | 13.39 ± 4.24 | 11.93 ± 3.03 | 0.729 |
| nLDL | 5.38 ± 1.04^§^ | 18.04 ± 2.71 | 0.005 |
| agLDL | 6.99 ± 1.36^§^ | 21.55 ± 3.87 | 0.015 |
| 4 hours |  |  |  |
| control | 34.63 ± 4.38 | 29.17 ± 6.77 | 0.338 |
| nLDL | 14.32 ± 3.03^§^ | 33.18 ± 2.90 | 0.001 |
| agLDL | 15.33 ± 1.82^§^ | 44.84 ± 2.92 | 0.002 |
| 6 hours |  |  |  |
| control | 60.38 ± 7.20 | 38.69 ± 6.42 | 0.010 |
| nLDL | 23.18 ± 5.96^§^ | 49.68 ± 7.80 | 0.001 |
| agLDL | 26.65 ± 4.17^§^ | 58.82 ± 5.36 | 0.002 |
| 8 hours |  |  |  |
| control | 79.42 ± 5.33 | 59.90 ± 5.93 | 0.036 |
| nLDL | 35.34 ± 9.90^§^ | 66.72 ± 8.99 | 0.001 |
| agLDL | 34.41 ± 7.21^§^ | 76.00 ± 3.295 | 0.001 |
| 12 hours |  |  |  |
| control | 95.84 ± 1.21 | 83.27 ± 3.67 | 0.177 |
| nLDL | 48.04 ± 10.67^§^ | 86.84 ± 2.19 | 0.001 |
| agLDL | 46.37 ± 10.39^§^ | 92.92 ± 2.62 | 0.001 |

Rosuvastatin was used at a concentration of 10 μM. Values are given as mean±SEM (n=6 for each group). *Comparison between rosuvastatin treated *vs* non-treated VSMC was performed by the T-Student test. At each time period, statistical significance for group differences was calculated by the one-way analysis of variance (ANOVA) followed by the Fisher’s PLSD. ^§^ P<0.005 compared to the control group.

**
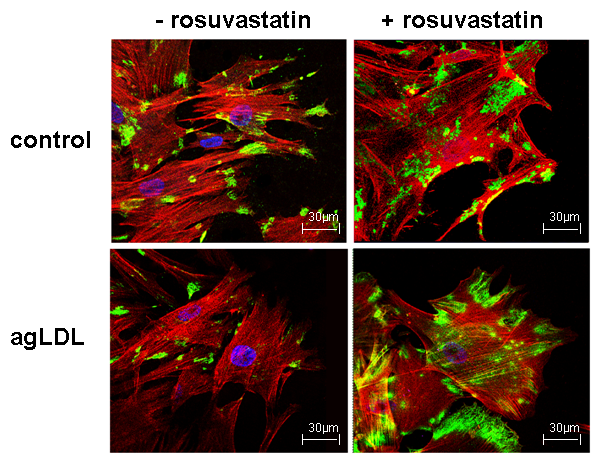
**

**Figure S1**

**Figure S2**


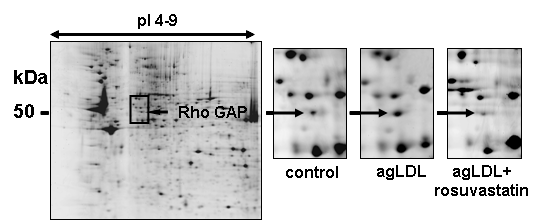


**Figure S3**
