# Supplementary material for: LDL-Induced Impairment of Human Vascular Smooth Muscle Cells Repair Function Is Reversed by HMG-CoA Reductase Inhibition
Source: PLoS One. 2012 Jun 12;7(6):e38935. doi: 10.1371/journal.pone.0038935 (PMC3373563; doi:10.1371/journal.pone.0038935)
Supplement: Table S2 — Effect of rosuvastatin (10 µM) on levels of [3H]thymidine incorporation in human VSMC exposed to 100 µg/mL nLDL or agLDL. (DOC) [file pone.0038935.s002.doc]

**Table S2**. **Effect of rosuvastatin (10µM) on levels of [^3^H]thymidine incorporation in human VSMC exposed to 100 µg/mL nLDL or agLDL**

|  | **- rosuvastatin** | **+ rosuvastatin** | **% decrease** | **P value*** |
| --- | --- | --- | --- | --- |
| nLDL | 2992 ± 215 | 662 ± 250 | 79.0 ± 6.3 | 0.001 |
| agLDL | 2686 ± 303 | 341 ± 170 | 80.0 ± 7.5 | < 0.001 |

Rosuvastatin was used at a concentration of 10 μM. Values are given as mean±SEM of counts per minute (CPM) (n=4 independent experiments performed in quadruplicates). *Comparison between rosuvastatin treated *vs* non-treated VSMC was performed by Mann Whitney test.
