# Supplementary figures and images for: LDL-Induced Impairment of Human Vascular Smooth Muscle Cells Repair Function Is Reversed by HMG-CoA Reductase Inhibition
Source: PLoS One. 2012 Jun 12;7(6):e38935. doi: 10.1371/journal.pone.0038935 (PMC3373563; doi:10.1371/journal.pone.0038935)

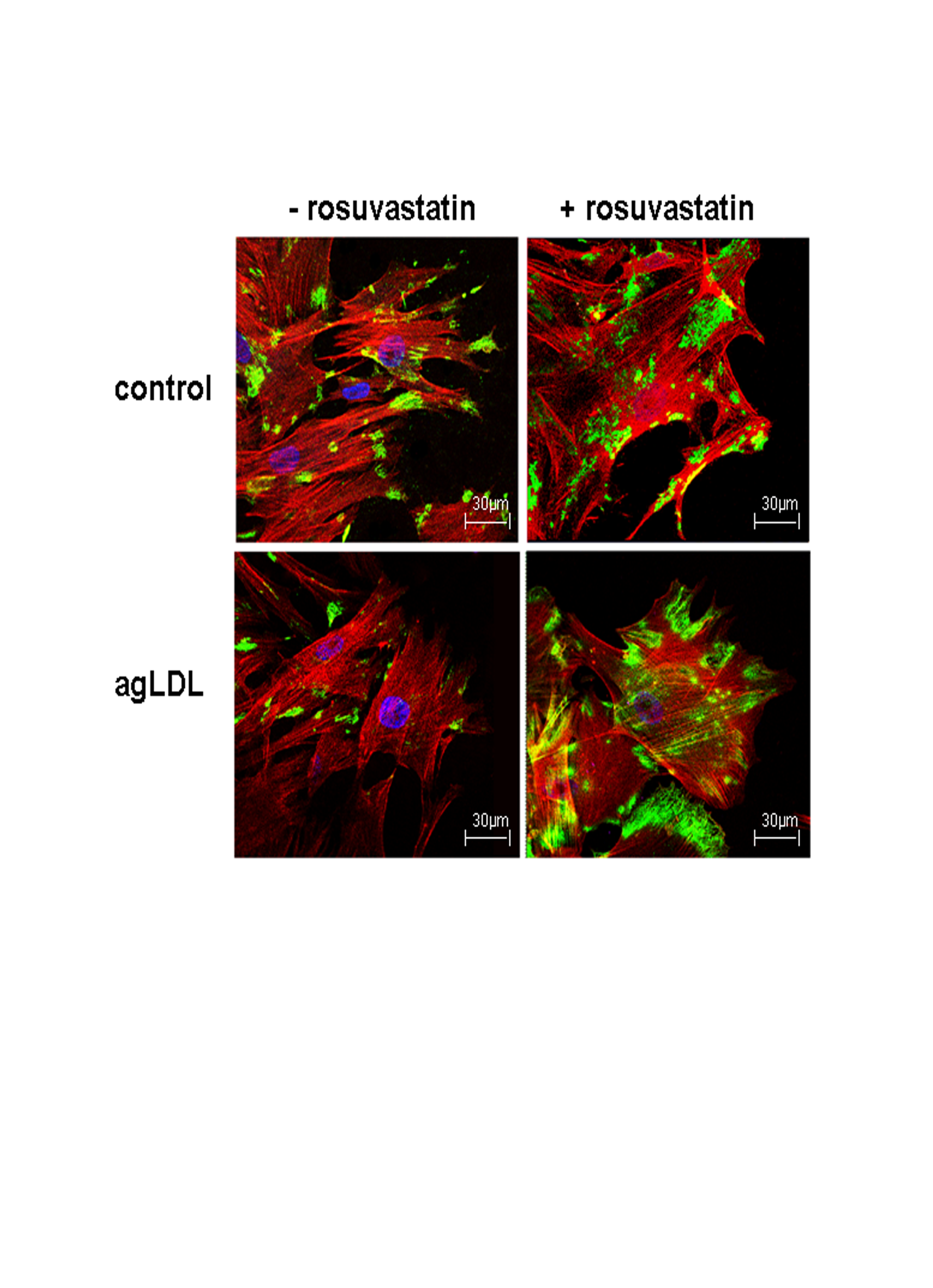

Supplement: Figure S1 — Effect of rosuvastatin on subcellular localization of phosphorylated MRLC in migrating VSMC during wound healing. Confocal microscopy of migrating cells (10%FCS-stimulated), 4 hours after wounding. Human coronary VSMC treated as shown for 16 hours. Cells were labelled for P-MRLC (Alexa Fluor 488-conjugated, signal in green) and F-actin (Alexa-Fluor 633 conjugated phalloidin, signal in red). (TIF) [file pone.0038935.s003.tif]

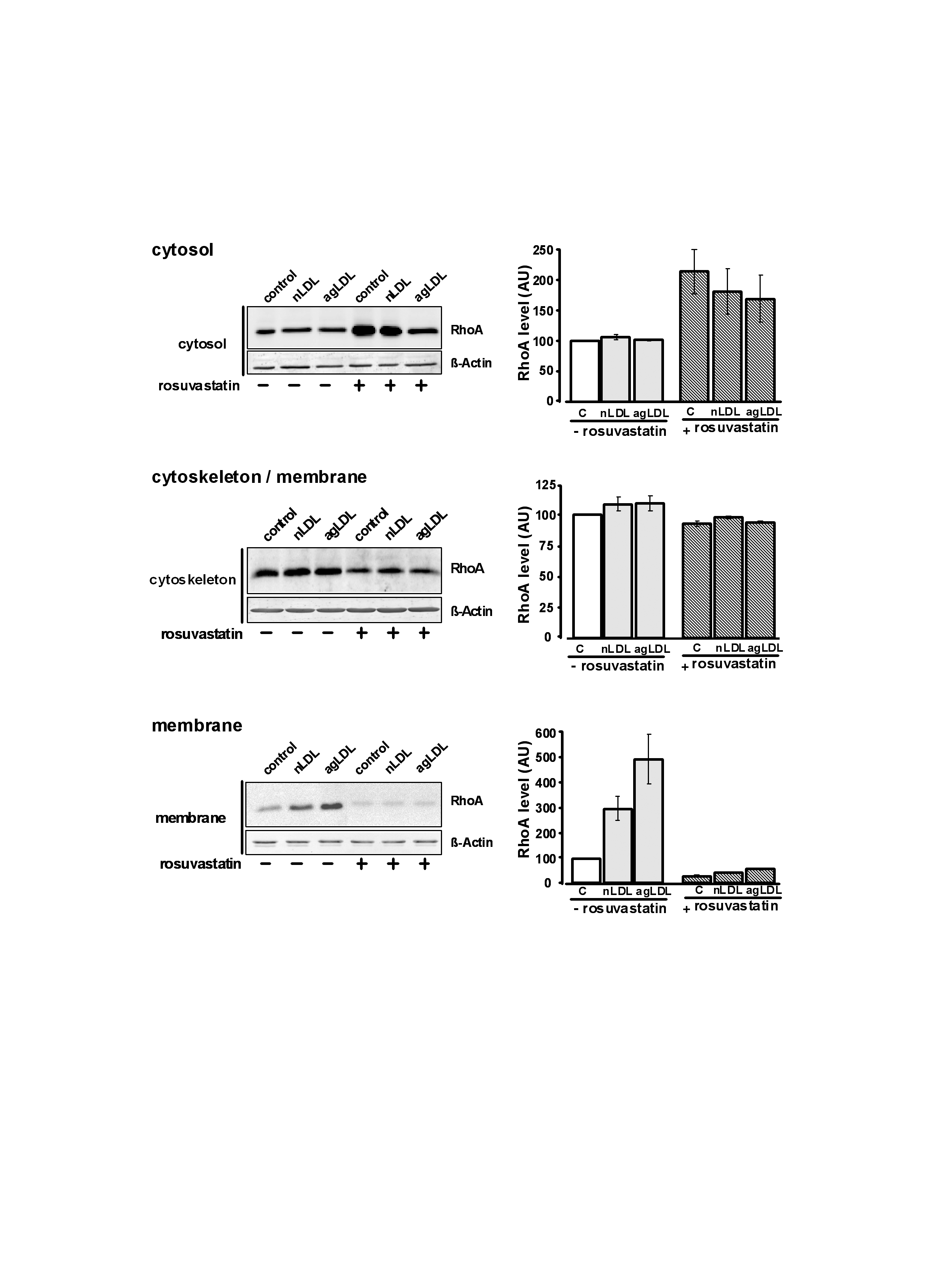

Supplement: Figure S2 — Increase of RhoA levels upon rosuvastatin treatment in the cytosol of human VSMC. RhoA and β-actin in cytosol, cytoskeleton-membrane and membrane extracts of VSMC with/without LDL and treatment with rosuvastatin or vehicle for 24 hours. The histograms show results expressed as % of the controls after normalization for β-actin. Results are given as mean±SEM of 3 independent experiments. (TIF) [file pone.0038935.s004.tif]

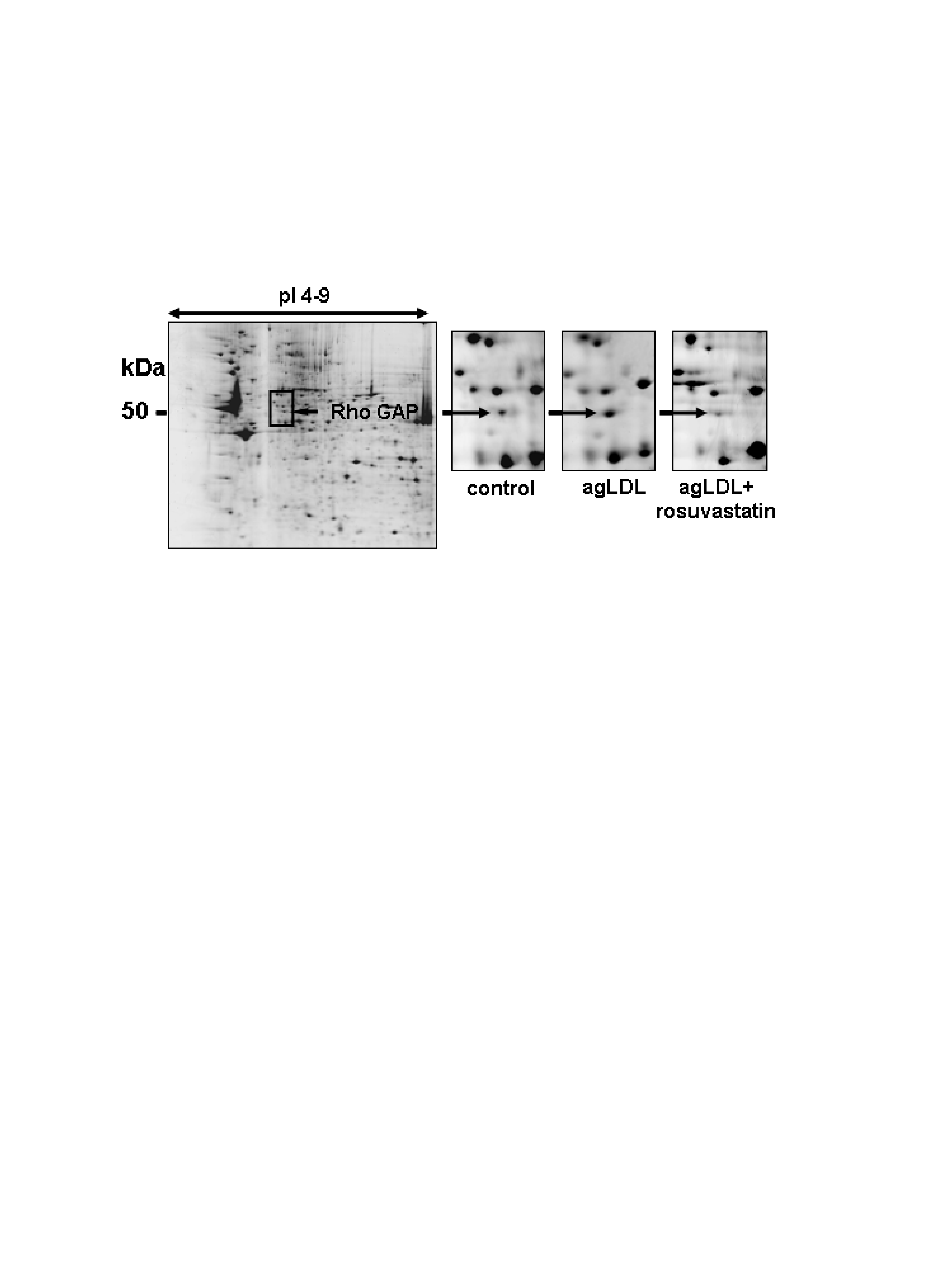

Supplement: Figure S3 — Rosuvastatin decreases p50RhoGAP level in LDL-treated VSMC. Representative 2-DE gel of the urea/detergent-soluble fraction of human VSMC. Enlarged images correspond to the gel area where p50RhoGAP (pI 5.9, MW 50 kDa) was detected. Rosuvastatin markedly decreases the labelling signal for p50RhoGAP (agLDL+rosuvastatin) in VSMC treated with agLDL. (TIF) [file pone.0038935.s005.tif]
